# Translocator protein in the rise and fall of central nervous system neurons

**DOI:** 10.3389/fncel.2023.1210205

**Published:** 2023-06-21

**Authors:** Garett Cheung, Yiqi Christina Lin, Vassilios Papadopoulos

**Affiliations:** Department of Pharmacology and Pharmaceutical Sciences, Alfred E. Mann School of Pharmacy and Pharmaceutical Sciences, University of Southern California, Los Angeles, CA, United States

**Keywords:** neurogenesis, neurodegeneration, mitochondria, ligands, 18 kDa translocator protein (TSPO)

## Abstract

Translocator protein (TSPO), a 18 kDa protein found in the outer mitochondrial membrane, has historically been associated with the transport of cholesterol in highly steroidogenic tissues though it is found in all cells throughout the mammalian body. TSPO has also been associated with molecular transport, oxidative stress, apoptosis, and energy metabolism. TSPO levels are typically low in the central nervous system (CNS), but a significant upregulation is observed in activated microglia during neuroinflammation. However, there are also a few specific regions that have been reported to have higher TSPO levels than the rest of the brain under normal conditions. These include the dentate gyrus of the hippocampus, the olfactory bulb, the subventricular zone, the choroid plexus, and the cerebellum. These areas are also all associated with adult neurogenesis, yet there is no explanation of TSPO’s function in these cells. Current studies have investigated the role of TSPO in microglia during neuron degeneration, but TSPO’s role in the rest of the neuron lifecycle remains to be elucidated. This review aims to discuss the known functions of TSPO and its potential role in the lifecycle of neurons within the CNS.

## Introduction

**FIGURE 1:**
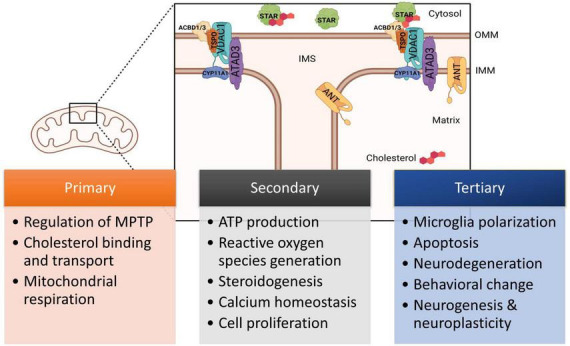
Diagram of TSPO and associated proteins located in the mitochondria demonstrating structural organization and the transport of cholesterol. Figure not drawn to scale. Currently known relationships with TPSO and downstream functions are indicated, including primary, secondary, and tertiary effects. ACBD1, acyl-CoA binding domain 1; ACBD3, acyl-CoA binding domain 3; ANT, adenine nucleotide translocator; ATAD3, ATPase family AAA domain-containing protein 3A; CYP11A1, cytochrome P450 11A1 cholesterol side-chain cleavage enzyme; IMM, inner mitochondrial membrane; OMM, outer mitochondrial membrane; STAR, steroidogenic acute regulatory protein; TSPO, translocator protein; VDAC1, voltage-dependent anion-selective channel 1. This figure created from biorender.com.

Translocator protein (TSPO) is an 18 kD five transmembrane-domain protein found in the outer mitochondrial membrane (OMM) and distributed throughout the mammalian body (Papadopoulos et al., 2006). This protein contains both cholesterol- and drug-binding domains and was previously known as the peripheral benzodiazepine receptor (PBR) due to its ability to bind the benzodiazepine diazepam (Marangos et al., 1982). TSPO forms a complex with voltage-dependent anion-selective channel 1 (VDAC1), spanning the outer mitochondrial membrane (Veenman et al., 2008; Gatliff et al., 2014), and interacts with several proteins, including ATPase family AAA domain-containing protein 3A (ATAD3), steroidogenic acute regulatory protein (STAR), acyl-CoA binding domain 1 (ACBD1), or diazepam binding inhibitor (DBI) and ACBD3 (Fan and Papadopoulos, 2013) to form complexes (Figure 1). It is most known for facilitating cholesterol transport from the cytosol into the mitochondria (Krueger and Papadopoulos, 1990; Papadopoulos et al., 2015; Biswas et al., 2018; Li et al., 2021). Studies have also supported its contribution to mediating ion transport and metabolites, oxidative stress, inflammation, apoptosis, and energy metabolism through downstream pathways (Stoebner et al., 2001; Santidrián et al., 2007; Veenman et al., 2008; Shargorodsky et al., 2012; Mendonça-Torres and Roberts, 2013).

Translocator protein contains a cholesterol-recognition amino acid consensus sequence (CRAC) (Li and Papadopoulos, 1998; Jamin et al., 2005; Jaipuria et al., 2017), ubiquitously found in transmembrane proteins that interact with cholesteryl groups (Vincent et al., 2002). Administration of a CRAC peptide prevents the opening of the mitochondrial permeability transition pore (MPTP) (Azarashvili et al., 2016). Both cholesterol and TSPO ligands have been shown to bind to MPTP with nanomolar affinity across species (Papadopoulos et al., 1992; Lacapère et al., 2001). Nevertheless, despite extensive study of TSPO’s facilitation of cholesterol transport into the mitochondria (Li and Papadopoulos, 1998; Li et al., 2001), recent knockout studies dispute this claim (Morohaku et al., 2014). A review of the alternative results along with all prior literature, suggests that the conservation of TSPO across all mammalian species increases the likelihood that redundant mechanisms are available to compensate for its removal (Papadopoulos et al., 2018), making the study of TSPO and its functions more complex.

Classically, TSPO’s high levels in steroidogenic organs have been associated with the production of steroids in the endocrine system (Papadopoulos et al., 1990, 2015). However, TSPO is found in cells all over the body. The central nervous system (CNS) normally expresses TSPO at low levels, making it notable when there is significant upregulation detected during neuroinflammation associated with dementia and other neurodegenerative diseases. Although higher levels of TSPO in the CNS are generally detected during inflammation, there are five specific regions in the adult brain where TSPO has been detectable under normal conditions. These include the olfactory bulb (OB), the subventricular zone (SVZ), the dentate gyrus of the hippocampus, the choroid plexus, and the cerebellum (Betlazar et al., 2018; Nutma et al., 2021). One common characteristic of these regions of the brain is that they are all associated with adult neurogenesis. While studies have examined the clinical application of TSPO, specifically with inflammation, only limited research has explored TSPO’s role in the neuronal life cycle. Currently, it is still unknown why TSPO is highly expressed in these brain regions. This review aims to discuss the possible function of TSPO in the life cycle of neurons, both directly and indirectly, through interactions with other cell types in the CNS.

## Current known functions of TSPO

Translocator protein was initially found in the pursuit of other proteins that benzodiazepines would bind to, and [^3^H]Ro5-4864 was used to characterize it (Marangos et al., 1982). As a result, Ro5-4864 (Ki: 1.02–20.04 nM) (Kita et al., 2004; Costa et al., 2016) was used as an exploratory treatment to determine the effect of ligand binding to the protein. Since then, other ligands have been developed in attempts to increase the binding specificity and affinity. Initial ligands included PK 11195 (Ki: 0.602–3.60 nM) (Kita et al., 2004; Costa et al., 2016) and FGIN-1-27 (Ki: 3.25 nM) (Kita et al., 2004). These were followed by XBD173 (Ki: 0.297 nM) (Kita et al., 2004), Etifoxine (Ki: 7.8–9.0 μM) (Costa et al., 2017; Bloms-Funke et al., 2022; Owen et al., 2022), SSR-180 575, and PIGA (N,N-Dialkyl-2-phenylindol-3-ylglyoxylamide; Ki: 0.31 to 343.01 nM) (Costa et al., 2016). Most recently GRT-X [(N-[(3-fluorophenyl)-methyl]-1-(2-methoxyethyl)-4-methyl-2-oxo-(7-trifluoromethyl)-1H-quinoline-3-caboxylic acid amide]; Ki: 0.07–4.60 μM) (Bloms-Funke et al., 2022), 2a (Ki: 40-200 nM) (Hallé et al., 2017), 2b (Ki: 5.5–6 nM) (Hallé et al., 2017), and ZBD-2 (N-benzyl-N-ethyl-2-(7,8-dihydro-7-benzyl-8-oxo-2-phenyl-9H-purin-9-yl) acetamide; Ki: 0.463 nM) (Wang D. et al., 2015) have also been used as TSPO ligands (Table 1). These ligands exhibit varying binding affinities depending on whether mitochondrial homogenates, cells or whole organ tissue was used for the assay. The introduction of these ligands allows for examining the function of TSPO when it is present. However, a genetic knockout or knockdown model can assess the basal biological function of TSPO and determine any compensatory mechanisms when the protein is removed from the system. These methods are not mutually exclusive and are important to be used in conjunction to fully understand the role of TSPO. This section outlines and explains the current known functions of TSPO.

**TABLE 1:** Summary of TSPO ligands and their observed effects within *in vitro* and *in vivo* models.

TSPO Ligand	Binding affinity	Steroidogenesis	Energy metabolism	Neurological disorders	Neuron development/repair	Inflammation	Apoptosis
PK 11195	Ki: 0.602–3.60 nM^27,28^	1 μM–↑ PREG, Y-1^24^ 100 nM–n.e.^44^ PREG, BV2 40 μM–↑^36^ PREG, C6 and U87MG	10 nM, 100 nM, 1 μM–↑^36^ OMAP index, U87MG	1 mg/kg–↓^143^ LPS-induced cognitive dysfunction	50 μM–↑^163^ differentiation, P19	3 mg/kg–↓^140^ neuroinflammation, mice 0.5 mM–↓^139^ pro-inflammatory cytokines, BV2	10 nM, 100 nM–↓^124^ DRG death
Ro5-4864	Ki: 1.02–20.04 nM^27,28^	1–50 μM–T, rat LC ↑^39^ 10 nM–n.e.^45^ SH-SY5Y 40 μM–↑^164^ PREG, SH-SY5Y	10 nM–↑ ATP^32^, SH-SY5Y	100 nM, 1 μM–↓^89^ β-amyloid toxicity, pN organoid	10 nM, 100 nM, 1 μM–↑^105^ neuron differentiation, neurite arborization, pN	5 mg/kg–↑^144^ anti-inflammatory factors and microglia M2 polarization	10 nM, 100 nM–↓^124^ DRG death 2.5, 10 mg/kg/day–↓ sciatic neuron death in neonatal mice
FGIN-1-27	Ki: 3.25 nM^28^	40 μM–↑^35^ T, pLC 0.01–50 μM–↑^39^ 1 mg/kg–↑^35^ T, rat serum		0.14, 0.28, 0.57, 1.1, 2.3 mg/kg–↓^93^ anxiety/depression, non-mammalian			100 μM–↑^66^ apoptosis, HT29
Etifoxine	Ki: 7.8–9.0 μM^29–31^	25,50 mg/kg–↑^40^ PREG/5α-DHP/3α,5α-THP, rat brain and plasma	50 mg/kg–↓^58^ OXPHOS impairment caused by mitochondria dysfunction	150 mg/day–↓^97^ HAM-A score, human	20 μM–↑^123^ neurite extension, PC12 50 mg/kg–↑^123^ axon regrowth, rat nerve	50 mg/kg–↓^141^ TBI induced inflammation, rat	25, 50 mg/kg–↓^63^ Caspase-3 during TBI, rat
SSR-180,575		10 nM–↑^45^ PREG, Co 20 μM^32^, 40 μM^32,164^–↑ PREG, SH-SY5Y	10 nM–↑ ATP^32,45^, SH-SY5Y		10 mg/kg–↑^62^ neuron repair		3, 6, 10 mg/kg–↑^62^ neuron survival
XBD173	Ki: 0.297 nM^28^	10 nM^45^, 20 μM^32^, 40 μM^164^–↑ PREG, SH-SY5Y	10 nM–↑^32,45^ ATP, SH-SY5Y	10, 20 mg/kg–↓^88^ MS clinical symptoms, mice 0.3, 1 mg/kg–↓ depression		10, 20 mg/kg–↓^88,148^ inflammatory cytokine, mice 20, 50 μM–↓ inflammatory cytokine, BV-2	10 mg/kg–↓^148^ retinal cell death, mice
PIGA	Ki: 0.31 to 343.01 nM^27^	10 μM–↑^47^ PREG, C6 40 μM–↑^36^ PREG, C6 and U87MG	10 nM to 1 μM–↑^36^ OMAP index, U87MG/NHA	30 mg/kg–↓^92^ anxiety in rats		3 μM–↓^165^ inflammatory IL-6, pPR	3 μM–↓^165^ Caspase-3, pPR
GRT-X	Ki: 0.07–4.60 μM^31^	10 μM–↑^127^ PREG, C6		0.316 to 10 mg/kg–↓ mechanical hyperalgesia^127^			5,10 mg/kg–↑ neuron survival/recovery in rat^127^
2a	Ki: 40-200 nM^32^	10 μM^32^, 20 μM^32^, 40 μM^32,164–^↑ PREG, SH-SY5Y	10 nM–↑ ATP^32,45^, SH-SY5Y				10 nM–↓ ROS and cellular death, SH-SY5Y
2b	Ki: 5.5-6 nM^32^	20 μM^32^, 40 μM^164^- ↑ PREG, SH-SY5Y	10 nM–↑ ATP^32,45^, SH-SY5Y				10 nM–↓ ROS and cellular death, SH-SY5Y
ZBD-2	Ki: 0.463 nM^33^			3 mg/kg/day–↓^90^ anxiety, mice 0.5,1.5 mg/kg/day–↓^33^ anxiety, mice			

PIGA, (N,N-Dialkyl-2-phenylindol-3-ylglyoxylamide); GRT-X, (N-[(3-fluorophenyl)-methyl]-1-(2-methoxyethyl)-4-methyl-2-oxo-(7-trifluoromethyl)-1H-quinoline-3-caboxylic acid amide); 2a, (imidazo[1,2-c]quinazolin-5-one derivative); 2b, (imidazo[1,2-c]quinazolin-5-one derivative); ZBD-2, [N-benzyl-N-ethyl2-(7,8-dihydro-7-benzyl-8-oxo-2-phenyl-9H-purin-9-yl) acetamide]; pLC, primary Leydig cell; pN, primary neuron; pPR, primary photoreceptor cells.

### Steroidogenesis

The most prominent role of TSPO in the periphery, steroidogenesis, has been extensively studied with data supporting the increase of steroid production through administration of TSPO ligands PK 11195, FGIN-1-27, PIGA, and Ro5-4864 (Papadopoulos et al., 1990; Chung et al., 2013; Da Pozzo et al., 2016; Chen et al., 2019; Table 1). Treatment of Leydig cells, the primary testosterone-producing cells in the male reproductive system, with diazepam binding inhibitor (DBI) increased steroid production (Papadopoulos et al., 1991; Garnier et al., 1994). DBI is an endogenous TSPO ligand isolated from rat brains. This increase in steroid production was also observed in rodent studies with adult rats treated with other TSPO ligands (Chung et al., 2013; Liere et al., 2017).

The involvement of TSPO in steroidogenesis is supported by genetic *TSPO* knockouts both *in vitro* and *in vivo*. However, recent studies have also challenged TSPO’s role in steroidogenesis from various knockout models (Morohaku et al., 2014; Tu et al., 2015). Nevertheless, additional studies utilizing CRISPR/Cas9-mediated knockout of TSPO in mouse Leydig cells showed a marked reduction in mitochondrial membrane potential and steroid formation (Fan et al., 2018). Similar steroidogenic abnormalities were also observed in TSPO knockout mice and rats, where total steroid production was decreased, and an increase in steroidogenic flux was observed (Owen et al., 2017; Barron et al., 2018).

It should also be noted that studies challenging the role of TSPO in steroidogenesis with TSPO knockout models observed the highest steroid production at drug concentrations 10–100 times greater than previous publications. The most noticeable increase occurred at 100 times higher concentrations; however, it is not certain if this increase was significant since the statistics were not published (Tu et al., 2015). This increase could be attributed to off-target binding of the ligand used (PK 11195), as it was given at a much higher concentration than needed for observable steroidogenic effects found in previous studies.

In addition to the effect on peripheral steroidogenesis, TSPO ligands have been reported to increase steroid production within the CNS as well. Ligands XBD173 and Ro5-4864 increased pregnenolone synthesis in the BV-2 microglia cell line (Bader et al., 2019). The same effect was observed when TSPO ligands were used to treat human neuroblastoma SH-SY5Y cells (Hallé et al., 2017; Lejri et al., 2019), human microglia cell lines (Da Pozzo et al., 2016; Lin et al., 2022), human astrocyte cell line (Lin et al., 2022), human glioblastoma cells (Lin et al., 2022), and rat glioma C6 cells (Da Pozzo et al., 2016; Santoro et al., 2016). Along with *in vitro* studies, TSPO ligands XBD173, SSR-180,575, PK 11195, and FGIN-1-27 were found to increase brain neurosteroid levels *in vivo* as well (Rupprecht et al., 2009; Porcu et al., 2016). These data show that TSPO, though barely expressed in the CNS under normal conditions, is still present and functional in the production of neurosteroids.

### Energy metabolism

Translocator protein influences energy metabolism in the mitochondria, specifically the membrane potential and oxygen consumption rate, playing a role in cellular respiration. TSPO has been shown to also interact with the MPTP, consisting of VDAC1 and the adenine nucleotide translocator (ANT), and contributes to the generation of reactive oxygen species (ROS), moderation of Ca^2+^ homeostasis, and ATP production (Veenman et al., 2008; Banati et al., 2014; Gatliff et al., 2014, 2017; Papadopoulos et al., 2015; Biswas et al., 2018; Farhan et al., 2021). Though there has been contention with the interaction of TSPO, VDAC1, and ANT as the MPTP opened in the absence of these proteins, evidence suggests that they are still involved potentially with the MPTP pore formation and in the apoptosis cascade (Veenman et al., 2008; Camara et al., 2017; Shoshan-Barmatz et al., 2019). Previous studies reporting changes in mitochondrial membrane potential have found altered ATP production (Vojtíšková et al., 2004) and oxygen consumption rates (Schuchmann et al., 2005) *in vivo* and *in vitro* (Zorova et al., 2018). TSPO deficiency in fibroblasts exhibited a decrease in oxygen consumption rate and mitochondrial membrane potential (Zhao et al., 2016). Similarly, microglia isolated from TSPO knockout animals had lower ATP synthesis (Banati et al., 2014), while TSPO insertion into human leukemia cell lines resulted in increased cellular excitability and ATP production (Liu et al., 2017). TSPO ligands had a similar effect in improving ATP production impaired by mitochondrial injury (Palzur et al., 2021).

Additionally, TSPO is involved in mitochondrial respiration within the CNS. An increase in ATP synthesis was observed when human neuroblastoma SH-SY5Y cells were treated with TSPO ligands (Lejri et al., 2019; Grimm et al., 2020). A reverse effect was observed with a CRISPR-Cas9 TSPO knockout, decreasing cellular respiratory function and mitochondrial membrane potential (Milenkovic et al., 2019). The impaired mitochondrial functions were rescued with lentiviral overexpression of TSPO in the knockout cells. As a result, it appears that TSPO in the CNS may be responsible for both overall mitochondrial health and ATP production.

Translocator protein has also been reported to play a role in apoptosis and has been investigated as a possible treatment in oncology. Depending on the stage of breast cancer progression, changes in TSPO have both proliferative and decreased viability effects (Wu and Gallo, 2013). An increase in TSPO has also been linked to both increases in expression and gene amplification likely part of a feedback system with ROS induction (Batarseh and Papadopoulos, 2010). Interactions with VDAC1 may also be a way TSPO contributes to apoptosis. TSPO ligands PK 11195 and Ro5-4864 altered MPTP opening and were shown to inhibit the pro-apoptotic function of erucylphosphohomocholine (an alkylphosphocholine antitumor agent) at high concentrations, preventing the collapse of the mitochondrial membrane potential (Veenman et al., 2008). TSPO ligands have also been shown to increase cell survival (Ferzaz et al., 2002) and decrease Caspase-3 apoptotic cells (Shehadeh et al., 2019). This was further supported with studies showing that the downregulation of TSPO expression inhibited oxidative stress from anoxia/reoxygenation insult (Meng et al., 2020). Treatment with TSPO ligands PIGA also protected photoreceptor-like cells from damage induced by lipopolysaccharide (LPS)-driven inflammation, resulting in a noticeable increase in cell viability at nanomolar doses (Corsi et al., 2022). On the other hand, one paper found that treatment with ligand FGIN-1-27 induced apoptosis in HT29 cancer cells and correlated with decreased membrane potential, while ligand PK 11195 induced apoptosis through Bcl-2 downregulation (Maaser et al., 2005). Nevertheless, there is little understanding of how TSPO may be influencing viability and regulated cell death. Possible explanations include altering the mitochondrial membrane potential or modulation of ROS, leading to apoptosis.

Reactive oxygen species are essential for cell regulation and are highly involved with cellular signal transduction, particularly in metabolism and apoptosis. ROS are byproducts of mitochondrial respiration but are also found altered in disease and cause proinflammatory cytokine release (Bulua et al., 2011). Oxidative stress leads to elevated inflammation, which is associated with cell death and apoptosis. Specifically, ROS play a role in LPS-driven inflammation caused by cytokine release (Bulua et al., 2011). Injury caused by anoxia/reoxygenation injury in cardiomyocytes has also resulted in increased TSPO expression and subsequent increased oxidative stress (Meng et al., 2020). On the other hand, downregulation of TSPO expression with RNA interference or the administration of various TSPO ligands has been found to decrease oxidative stress (Biswas et al., 2018; Meng et al., 2020). Additional studies have also reported the inhibition of TSPO by FGIN-1-27-amended Ca^2+^ overload caused by chemical ischemia in a cardiac cell line (Li et al., 2010). As a result, TSPO can alter ROS production, influence the signaling cascade, and lead to expedited progression of disease.

### Neurological disorders

Translocator protein has been closely tied to neurodegenerative diseases like Alzheimer’s Disease (AD) and Parkinson’s Disease (PD) (Mcgeer et al., 1988; Zimmer et al., 2014; Edison et al., 2018; Zhou et al., 2021), due to the presence of dysfunctional mitochondria, and the increased inflammation as the innate response to degradation. AD and PD have both been linked to dysfunctional mitochondria, and TSPO has been reported to be capable of modulating mitochondrial health and mitophagy (Gatliff et al., 2014, 2017; Wang et al., 2020; Frison et al., 2021). Previous publications have also reported that the overexpression of TSPO led to impaired mitophagy, similar to those seen in PD and amyotrophic lateral sclerosis (ALS) (Frison et al., 2021; Magrì et al., 2023). This altered mitophagy and increased TSPO levels were also observed when mitochondrial respiration was significantly reduced. It is hypothesized that dysfunctional mitochondria may be involved in the progression of neurodegeneration, but the cause of the dysfunction remains to be elucidated.

The association between neurodegeneration and TSPO is also linked to the activation of microglia, the CNS’s resident macrophages. As a result, TSPO ligands have been used as fluorescent PET markers for inflammation in diagnosing AD and PD (Dheen et al., 2007; Karlstetter et al., 2014; Hamelin et al., 2016; James et al., 2017; Yokokura et al., 2017; Janssen et al., 2018; Daniela Femminella et al., 2019; Werry et al., 2019). Confusingly, the binding of [^11^C]PK 11195 failed in some patients known to have neuroinflammation. This has been attributed to the reduced binding affinity of [^11^C]PK 11195 to TSPO caused by a common polymorphism, where the 147th amino acid alanine is replaced with a threonine (Berroterán-Infante et al., 2019). New fluorescent TSPO ligands have been developed to bind regardless of the polymorphism (Vettermann et al., 2021). This polymorphism has been shown to have a 30% prevalence in individuals with European ancestry (Kreisl et al., 2013) and influences diurnal cortisol rhythm in individuals diagnosed with bipolar disorder (Prossin et al., 2018). The mutation not only affects ligand binding but also alters the binding of cholesterol to the CRAC domain (Li et al., 2015). On the other hand, this polymorphism has been shown to have no effect on the endogenous TSPO ligand DBI (Berroterán-Infante et al., 2019). These findings indicate that mutations in TSPO can have far-reaching consequences with endocrine signaling and lead to behavior alterations.

Translocator protein ligands have also been studied for their potential effects on limiting neurological disorders. PK 11195 was found to improve AD-related behavior and β-amyloid pathology in 3xTg-AD rats (Christensen and Pike, 2018), and XBD173 improved clinical symptoms of multiple sclerosis (MS) (Leva et al., 2017). Ro5-4864 also showed protection against β-amyloid toxicity in organotypic hippocampal cultures (Arbo et al., 2017a). However, the mechanism by which TSPO affects neurodegenerative disorders remains to be elucidated.

The anxiolytic and anti-depressive effects of TSPO ligands have been investigated as an alternative to conventional benzodiazepines. An anxiolytic effect was seen in chronic unpredictable mild stress-conditioned mice when TSPO ligand, ZBD-2 [N-benzyl-N-ethyl2-(7,8-dihydro-7-benzyl-8-oxo-2-phenyl-9H-purin-9-yl) acetamide], was administered (Wang et al., 2017). This effect was prevented by the coadministration of PK 11195. Administration of ZBD-2 also decreased depression in the same mice and depressive behavior in mouse models of postpartum depression (Li et al., 2018). This ligand was also reported to decrease anxiety-like behaviors caused by chronic inflammatory pain (Wang D. et al., 2015). Similar results were observed with an elevated-plus maze anxiety test in rats treated with PIGA (da Settimo et al., 2008). Anti-depressive effects were also seen with the administration of TSPO ligands FGIN-1-27 in non-mammalian models (Lima-Maximino et al., 2018) and XBD173 in rodent models of chronic stress and depression associated with diabetes mellitus (Qiu et al., 2016; Shang et al., 2020). It is believed that TSPO ligands alter neurological function by increasing the production of neurosteroids, which can act as inhibitory neurotransmitters (Longone et al., 2011). Neurosteroids have similarly been closely tied to the anxiolytic effects of TSPO, with ligands etifoxine being approved for treating anxiety (Nguyen et al., 2006) and brexanolone, the aqueous formulation of allopregnanolone, for treatment of postpartum depression (Scarff, 2019). Despite TSPO’s effect on anxiety and depression, the exact mechanism by which it acts is still unknown.

## TSPO role in neurons

**FIGURE 2:**
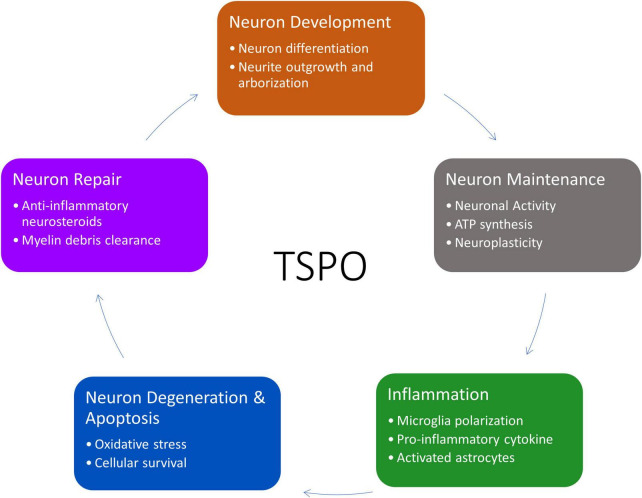
Overview of TSPO’s involvement in the life cycle of neurons from neurogenesis to apoptosis and recycling. This figure created from biorender.com.

Although the functions of TSPO have been extensively studied in the periphery, it remains to be elucidated in neurons. The current knowledge of TSPO functions (such as steroid production, energy, and apoptosis) could be used as a basis to theorize and explore the potential means that TSPO can affect the lifecycle of neurons, either directly or indirectly (Figure 2).

### Neurogenesis

Translocator protein has been difficult to detect in mature neurons, particularly in the normal brain. Recent studies have pointed toward a role for TSPO in neurogenesis, particularly as the few CNS regions with high TSPO are associated with neurogenesis. This was demonstrated by the discovery of TSPO in neural stem cells (NSC) by Varga et al. (2009). Moreover, the endogenous TSPO ligand DBI increased stem cell growth and expansion in neurogenesis (Dumitru et al., 2017), further indicating that TSPO may play a role in stem cell proliferation and differentiation. However, it may be related to GABA receptor function, as DBI can bind to GABA receptors (Bormann, 1991) and modulate GABA signaling (Everlien et al., 2022).

The role of TSPO in neurogenesis has also been investigated in neuronal differentiation with experiments using embryonic stem cell lines. Previous studies have found that the TSPO ligand PK 11195 could induce neuronal lineages. In PC12, a rat cell line of embryonic neural crest origin, PK 11195 treatment induced the development of neurites and increased expression of β-III tubulin, the neuron-specific tubulin in PC12 cells, without any additional supplements (Yasin et al., 2017). A study with N1E-115 cells, a mouse neuroblastoma cell line, also showed an increase in neuron-like morphology, but the cells were not tested for other neuronal markers (Krestinina et al., 2017). When PK 11195 was administered to P19 cells, a mouse embryonic carcinoma cell line, all neuronal markers were reported to increase, though not to the same level as cells treated with retinoic acid, the traditional cytokine used for differentiation (González-Blanco et al., 2021). Other TSPO ligands have also been shown to increase rates of neuron differentiation and neurite arborization in *ex vivo* models (Arbo et al., 2017b).

Although it appears that TSPO is directly involved in neurogenesis, different authors came to different conclusions. One study determined that the effect was not a result of TSPO but instead with mitochondrial 2′,3′-cyclo-nucleotide-3′-phosphodiesterase (Krestinina et al., 2017), while the other two studies either state that TSPO was directly involved in neuronal differentiation (González-Blanco et al., 2021) or that it indirectly led to differentiation through modulation of gene expression (Yasin et al., 2017). It should be noted that these experiments utilize only embryonic stem cells and cell lines. Studies have shown that genes and growth factors can have varying roles and effects depending on age and stage of development (Urbán and Guillemot, 2014). For example, γ-aminobutyric acid (GABA) promotes proliferation of embryonic NSCs (Haydar et al., 2000) but reduces proliferation in adult NSCs (Liu et al., 2005). As a result, TSPO’s function in stem cell survival and differentiation in adults remains to be elucidated.

### Neuronal maintenance

As neurogenesis is not limited to the developmental period of organisms, TSPO may be involved in adult neurogenesis as well. Due to the high energy requirement of differentiation, TSPO and its interaction with cellular respiration could be important in modulating energy metabolism and ATP production. In adult hippocampal neurons, an increase in TSPO was found in response to increased neuronal activity (Notter et al., 2020). The upregulation of TSPO could also be involved in the increased cellular respiration and ATP production needed for the high energy demands of action potential generation (Aiello and Bach-Y-Rita, 2000; Berndt and Holzhütter, 2013).

Translocator protein may also contribute to the control of neurogenesis in adults through modulation of apoptosis in mammalian adult brains. In the adult, neural stem cells created through neurogenesis are not all incorporated into the neural network, and less than 2% of all mature neurons are replaced regularly (Ryu et al., 2016). In the rat, dentate gyrus, over 50% of all neural stem cells generated through adult neurogenesis will undergo apoptosis before they develop into mature neurons (Cameron and Mckay, 2001; Dayer et al., 2003). Similarly, only 40% of mature cells from adult neurogenesis integrate into the olfactory bulb (Winner et al., 2002). As TSPO has been linked to programmed cell death in other areas of the body, the relationship of TSPO with adult neurogenesis may also reflect this linkage. However, the exact role of TSPO in neural stem cell apoptosis has not been explored.

Maintenance of developing neurons is beneficial to overall cognitive health, but limiting the degradation of existing mature neurons may prevent additional problems as well. TSPO overexpression in the hippocampus, achieved through lentiviral transfection, reduced cognitive impairments caused by LPS-induced inflammation in mice (Wang et al., 2016). This indicates that TSPO may play a neuroprotective role by modulating apoptosis, as seen in other examples in the periphery. Though there have been data published on the presence of TSPO in neurons and predicted explanations, the role of TSPO in neurons remains to be fully established.

Another possibility is that the presence of TSPO in neurons is related to its anxiolytic effect, specifically in the management of anxiety and neuroplasticity. Overexpression of TSPO in the hippocampal dentate gyrus resulted in anxiolytic effects in a post-traumatic stress-disorder mouse model (Zhang et al., 2018). However, it remains to be seen if this is a result of increased rewiring neurons in the memory processing centers of the brain or of TSPO’s direct anxiolytic effect discussed above. Neurosteroids have been shown to modulate GABA receptors (Akk et al., 2001; Hosie et al., 2007) and improve learning and memory in rodents (Marx et al., 2009). As a result, the effects of TSPO in neurons may be due to neurosteroid production and metabolism.

### Neuronal repair

Translocator protein may also be important in repair and regeneration, though most effects of TSPO ligands on neurons have primarily been studied in the peripheral nervous system (PNS). The TSPO ligand Ro5-4864 improved repair and remyelination of PNS neurons along with elevating brain-derived neurotropic factor (BDNF) levels in the dorsal root ganglion and sciatic nerve (Ma et al., 2018). However, a separate study using the same ligand found that TSPO did not alter BDNF levels between treatment conditions (Palzur et al., 2016). Therefore, it is not certain through which mechanism Ro5-4864 acts to promote recovery. Ro5-4864, along with etifoxine, was also shown to improve peripheral nerve injury repair and regeneration (Girard et al., 2008; Mills et al., 2008). Moreover, Ro5-4864 protected neonatal dorsal root ganglion neurons from apoptosis, though it had limited success in improving survival of mature neurons (Mills et al., 2008). Etifoxine also improved neurite outgrowth in PC12 cells, a cell line derived from a pheochromocytoma of the rat adrenal medulla (Girard et al., 2008). Thus, TSPO may play a role in neonatal development and survival of neural progenitors and might facilitate the repair of adult peripheral neurons.

Interestingly, all effects on neurons seen with TSPO ligands have been found in the PNS or in neurons taken during embryonic development. However, neurons in the PNS are capable of spontaneous regeneration after injury, while CNS neurons fail to do so (Huebner and Strittmatter, 2009). On the other hand, previous studies have shown CNS neuron regeneration when a PNS nerve graft was placed over them, indicating that CNS neurons do have the capacity to regrow when placed in a supportive environment (Stouffer et al., 1980). A recent study utilized another TSPO ligand GRT-X (N-[(3-fluorophenyl)-methyl]-1-[2-methoxyethyl]-4-methyl-2-oxo-[7-trifluoromethyl]-1H-quinoline-3-caboxylic acid amide), which is also an agonist for the voltage-gated potassium channel of the Kv7 family, to treat chronic neuropathic pain caused by traumatic injury (Bloms-Funke et al., 2022). It was effective in promoting the survival and repair of sensory and motor neurons. However, the injury in this study was also in the periphery, distal to the dorsal root ganglions, and indicates that TSPO ligands can influence peripheral nerve regeneration, but it did not explore any effects on the CNS. Alternatively, a study utilizing Ro5-4864 and its effect on traumatic brain injury in rats showed that TSPO protein was not detectable in control neurons but appeared to be upregulated in injured neurons (Palzur et al., 2016). This study confirmed the presence of TSPO during neuronal injury but again did not explore its function. As a result, the role of TSPO within CNS neurons remains to be elucidated despite the presence of the protein in individual CNS niches.

## TSPO in glial regulation of neuronal health

The study of TSPO in the CNS has primarily focused on neuroinflammation and neurodegeneration, primarily the involvement of microglia. Microglia are unique brain macrophages that differ in roles and functions from peripheral macrophages. Specifically, microglia act as the first line of defense within the CNS and respond to calcium waves (Olson and Miller, 2004; Sieger et al., 2012), while peripheral macrophages do not. Microglia respond to brain-damage Ca^2+^ and ATP signals, T helper cytokines, and LPS, activating to produce pro-inflammatory cytokines and inducing an M1 polarization (proinflammatory) state (Sieger et al., 2012; Franco and Fernández-Suárez, 2015). Microglia are also crucial for neuronal repair as they remove damaged cells and debris via phagocytosis to prepare the area for regeneration (Liu et al., 2012; Cai et al., 2019; Berghoff et al., 2021). In the event of injuries to the CNS, common inflammatory cytokines and ROS are released to initiate wound repair.

Translocator protein is involved in the phagocytotic ability of microglia. Microglia taken from TSPO knockout mice had impaired phagocytosis, which led to the accumulation of β-amyloid that was not properly cleared and removed (Zhang et al., 2021). This may be a result of the high energy demand of cytoskeletal reorganization during phagocytosis, where a decrease in energy production was accompanied by impaired phagocytosis (Yao et al., 2020; Fairley et al., 2023). These studies suggest that a shift toward glycolysis caused by TSPO knockout could lead to lower ATP production. This was also supported in experiments using TSPO knockout cells, TSPO knockout animals, and reduced TSPO levels found in aging animals (Garza et al., 2022). Alternatively, a shift toward glycolysis would also increase lactate production and lower pH levels. Cellular pH has been shown to alter the ability of macrophages to phagocytose (Grinstein et al., 1991). Moreover, modulation of microglia polarization via TSPO can also affect phagocytosis, as TSPO induces M1 polarization over M2. M2 polarization increases the production of scavenger receptors for phagocytosis and is more effective in its ability to uptake and degrade β-amyloid than M1 microglia (Cherry et al., 2014). Therefore, TSPO appears to be involved in the polarization of microglia required for phagocytosis, which is needed to remove apoptotic neural stem cells in the neurogenic niches. TSPO ligands have been found to reduce inflammation and β-amyloid generation *in vitro* (Xue et al., 2022) and *in vivo* (Ma et al., 2016; Simon-O’Brien et al., 2016) and reduce quinolinic-acid-induced degeneration (Leaver et al., 2012) and LPS-induced cognitive dysfunction in rats (Lan et al., 2021). Previous studies have also reported that TSPO ligands direct microglia toward M2 polarization to promote anti-inflammatory cytokines (Zhou J. et al., 2020) and increase microglia phagocytotic ability (Fairley et al., 2023).

Microglia activation increases cytokine release and is involved in the phagocytosis of cellular debris (Lampron et al., 2015). This response initiates the clearing of degraded myelin, leading to neuron recovery and repair (Lloyd et al., 2019; Berghoff et al., 2021). Microglia are also responsible for secreting anti-inflammatory cytokines to promote regeneration after removing the source of inflammation. This may also explain the reduction of β-amyloid accumulation with Ro5-4864 treatment (Barron et al., 2013), as it may increase microglia clearance of debris. The protective effects of TSPO ligands have also been investigated as possible treatments for neurodegenerative diseases, primarily by inhibiting the inflammatory response (Karlstetter et al., 2014; Scholz et al., 2015; Leva et al., 2017). For example, treatment with etifoxine reduced clinical pathology in a mouse model of MS by lowering the number of peripheral immune cells infiltrating the CNS and promoting remyelination after peripherally induced demyelination (Daugherty et al., 2013).

Translocator protein-modulated microglia polarization and neurogenesis are also connected via cytokines secreted by microglia that aid in the removal of apoptotic cells and promote neuron differentiation, depending on their polarization state. Initial pro-inflammatory activation of microglia to the M1 state is required for myelin clearance after demyelination (Cunha et al., 2020). M1 polarization further produces pro-inflammatory cytokines (Wang W. Y. et al., 2015) and increases ROS production necessary for macrophage myelin phagocytosis (Van Der Goes et al., 1998). Once there is sufficient breakdown, microglia shift to M2 state through anti-inflammatory signaling (Zhou D. et al., 2020; Guo et al., 2022). They can also secrete anti-inflammatory cytokines to increase the signal as well as begin phagocytosis and breakdown of myelin debris (Tang and Le, 2016; Guo et al., 2022). TSPO’s role in modulating the pro-inflammatory M1 and anti-inflammatory M2 polarization of microglia (da Pozzo et al., 2019) directly impacts the degradation and clearance of myelin and cellular debris that inhibits neurogenesis and promotes the regeneration process.

Translocator protein may also influence neuronal development via its function in glial cells. *De novo* neurosteroid synthesis can play a role in both the development and regulation of neuronal health and function and is primarily observed in glial cells. Progesterone directly influences neurogenesis, as progesterone treatment can increase the number of dopamine neurons during neuronal differentiation from mouse embryonic stem cells (Díaz et al., 2009). Similar effects were observed with human umbilical cord mesenchymal stem cell differentiation into neuron-like cells (Wang et al., 2012). *De novo* synthesized progesterone also increased dendritic spine formation in developing rat Purkinje cells (Sakamoto et al., 2002). Moreover, not only are neurosteroids involved in development, but they are also neuromodulatory. They can interact with extracellular GABA and potentiate GABA receptors on synaptic terminals. Specifically, progesterone, allopregnanolone, and deoxycorticosterone have been shown to promote the activation of GABA (Kokate et al., 1994; Reddy and Rogawski, 2002). Given the neurosteroid effects on neurogenesis and cognitive processes, in addition to TSPO’s demonstrated function in steroidogenesis, TSPO’s role in the CNS may include neurosteroid production used for the regeneration and maintenance of neurons.

## Conclusion

Translocator protein has long been studied in the PNS, particularly its involvement in steroidogenesis and energy metabolism, but few studies have delved into its function in the development and regulation of cells in the central nervous system. It is important to determine if the effects seen in the PNS are applicable to the CNS, as the behavior of cells varies depending on the environment and location. This is particularly evident in neurogenesis, where only specific areas within the adult brain are involved, and neuronal repair and regrowth, which occurs spontaneously in the PNS but not the CNS. Crucially, the CNS areas with significant neurogenesis are also where TSPO has been reported at higher levels than the rest of the CNS. While most neurogenic studies have focused on embryonic development, more investigation is needed to determine if factors affecting neurogenesis stay consistent throughout the development of the neural system into adulthood. TSPO has been shown to be involved in the development of cells but is also important in controlled cell death, repair, and regeneration of neurons (Figure 2). Further studies investigating the detailed involvement of TSPO in each of these functions specific to CNS neurons may provide more clarity on how it affects neuronal development and health, adding to our understanding of neuron degradation and turnover.

## Author contributions

GC, YL, and VP: conceptualization and writing–review and editing. GC: writing–original draft. VP: funding acquisition. All authors contributed to the article and approved the submitted version.
